# Objectively Measured Life Space Using Participants’ Mobile Phones: Cross-Sectional Study

**DOI:** 10.2196/73128

**Published:** 2026-09-21

**Authors:** Michelle C Odden, Annabel X Tan, Sylvie Dobrota Lai, J David Rhodes, Suzanne Judd, Kyle Sargent, Sheila Mwanda, Sage Rowe, Jiajun Wu, Abby C King, Vishnu Ravi

**Affiliations:** 1 Department of Epidemiology and Population Health, Stanford University, 1701 Page Mill Road, Stanford, CA, 94305, United States, 1 (650) 721-0230; 2 Department of Biostatistics, School of Public Health, University of Alabama at Birmingham, Birmingham, AL, United States; 3 Department of Computer Science, Stanford University, Stanford, CA, United States; 4 Stanford Prevention Research Center, Department of Medicine, Stanford University, Stanford, CA, United States; 5 Byers Center for Biodesign, Stanford University, Stanford, CA, United States

**Keywords:** mobile health, life space, aging, GPS tracking, built environment, mHealth

## Abstract

**Background:**

Measurement of life space characterizes people’s engagement with their environment. Several studies have used GPS devices to capture life space. A challenge with device-based measures is that participants may forget to bring the device with them. Life-space measures that leverage the GPS data from participants’ mobile phones may address this challenge, but few studies have leveraged this approach to date.

**Objective:**

Our objective was to assess the feasibility of implementing a mobile phone life-space assessment and characterize life space using Google Street View (GSV) imagery. We then aimed to evaluate the association of demographic data with life-space size and environmental features.

**Methods:**

We developed a mobile phone app to passively capture life space in older adults and sampled GSV images from participants’ life space to characterize the environments where they spent their time. For this cross-sectional study, we recruited 82 participants (n=34, 41.5% men) aged 65 years and older (mean 74.5, SD 6.5 years) in 2023 to 2024 from the REGARDS (Reasons for Geographic and Racial Differences in Stroke) study. They were followed for 2 weeks and invited to complete nightly questionnaires on their phones. We measured features of the built environment that captured walkability (sidewalks, benches, and streetlights), neighborhoods (houses), and green space based on an image segmentation algorithm. Differences in life-space size and environmental features across participant characteristics were tested using the Wilcoxon rank sum test.

**Results:**

Among 82 participants, 76 (92.7%) completed all 14 days of data collection. Median daily maximum distance from home was 6.6 (IQR 1.3-15.5) km. Median distance traveled over 2 weeks was 477.1 (IQR 234.4-817.13) km. Adults younger than 70 years, women, Black participants, and those with income more than US $75,000 per year had larger life space than those aged 70 years and older, men, White participants, and those with income less than US $75,000 (154.5 vs 58.1 km^2^, *P*=.04; 102.9 vs 46.0 km^2^, *P*=.07; 179.3 vs 62.8 km^2^, *P*=.03; and 145.5 vs 25.5 km^2^, *P*=.001, respectively), although not all of these comparisons met the α=.05 level of statistical significance based on the Wilcoxon rank sum test. We found no association between demographic characteristics and features of the life-space built environment (*P*>.05 for all comparisons).

**Conclusions:**

In this feasibility study, we demonstrated that personal mobile phones could be used to passively track older adults’ life space based on a digital health app developed on an open-source platform. There was wide variation in objectively measured life space, and we found sociodemographic patterning of life-space movement. Objective measurement of life space with participants’ mobile phones is feasible and should be evaluated in larger samples to better capture the relationship between health and time spent in different environments. Integration of a life-space assessment into older adults’ daily lives may allow for the use of life space as a health measure.

## Introduction

The global population of adults aged 60 years and older is projected to be 1.4 billion by 2030, and 2.1 billion by 2050 [1]. Scalable measures to monitor health and functional decline in aging adults are needed. Life space, or where and how often a person travels, is an important predictor of health outcomes, including physical function, cognition, and mortality among older adults [2-5]. It serves as an important marker of social engagement, community participation, and physical activity [6]. Life space is commonly measured by questionnaire, which is subject to recall bias, especially among older adults with cognitive impairment [7]. As a result, several studies have turned to GPS devices to capture movement and mobility to more accurately and precisely capture community mobility among older adults [8]. However, a challenge with device-based measures is that they can create an additional burden for participants [9], and the participant may forget to bring the device with them, especially if the device is new and not already part of an individual’s routine. Participants’ own smartphones are devices already integrated into daily routines; thus, leveraging these devices to capture life space can eliminate additional burden while enabling passive, continuous tracking. Despite these advantages, few studies have leveraged this approach to date [10-12].

In addition to their mobility patterns, the environment in which people spend their time is an important determinant of health outcomes. The built environment can influence health by providing opportunities for health-enhancing behaviors such as physical activity and recreation [13-16]. However, much of the research on the built environment and health has focused on macroscale, geographic information system–based measures such as residential density, street connectivity, land use, and parks [17-19]. Environmental audits can assess the built environment and microscale features but can be time-consuming and subject to interrater reliability. More recently, AI methods have been combined with widespread georeferenced imagery, such as Google Street View (GSV) images, to assess features of the environment [20]. This method has been used in studies on green space, walkability, and other built environment features [21-23].

Despite evidence that both life space and the built environment influence health, existing research commonly treats these as separate domains. Traditional life-space questionnaires cannot capture environmental features encountered during daily travel, while built environment studies rely on residential addresses, overlooking the locations where older adults actually spend their time. When life-space assessment is combined with street view imagery analysis, this approach can simultaneously measure where people go and the environments they encounter [9]. This work could provide scalable insights into person-environment interactions that influence health and functional outcomes in aging adults.

In this feasibility study, we developed a mobile phone app to passively capture life space in older adults. We then measured features of the participants’ life space over 2 weeks and sampled GSV imagery from their life space to assess features of the environment in which they spent their time. This study was conducted within the REGARDS (Reasons for Geographic and Racial Differences in Stroke), a cohort study of Black and White adults residing in the United States. The REGARDS cohort provides an ideal population for this feasibility study given its racial and geographic diversity and the well-characterized demographic and health data of participants [24]. The objective of this study was to assess the feasibility of implementing a mobile phone life-space assessment and evaluate the association of demographic data with life-space outcomes. We hypothesized that older age and lower socioeconomic status (measured by education and income) would be associated with smaller life space.

## Methods

### Study Design

Participants for this cross-sectional study (REGARDS Life Space) were recruited from the parent REGARDS study. The REGARDS study enrolled 30,239 Black and White participants aged ≥45 years from the continental United States between 2003 and 2007. The primary objective of the REGARDS study was to better understand the reasons for the excess stroke mortality among Black persons and among those residing in the southeastern United States [24]. Exclusion criteria included race other than African American or Black or White, active treatment for cancer, medical conditions that would prevent long-term participation, cognitive impairment judged by the telephone interviewer, residence in or inclusion on a waiting list for a nursing home, or inability to communicate in English [24]. Data for the parent REGARDS study were collected from participants during 2 in-home exams (baseline: 2003‐2007; exam 2: 2013‐2016) and ongoing telephone follow-up interviews every 6 months. Participants for the present study (REGARDS Life Space) were recruited during these phone calls in 2023 to 2024.

Additional eligibility criteria for the current study included the following: (1) having an iPhone model 8 or later, (2) usually bringing their iPhone with them when they left the house, (3) knowing how to download an app, and (4) not being homebound. REGARDS participants were screened for eligibility and then invited to participate in the study.

### Recruitment

Participants were recruited for the current study in 2023 to 2024 using a convenience sampling method from the parent REGARDS study. They were initially screened for eligibility during the REGARDS 6-month follow-up calls. Participants who were eligible were then invited to participate in the current study by mail per the REGARDS ancillary study protocols. Individuals who responded to the mailer were included in the study. The target sample size was 100 participants for this feasibility study based on the available funding and staffing.

### Participant Demographics

Data on age, gender (woman or man), race (Black or White), education (less than high school, high school, some college, or college and above), income (<US $35,000, US $35,000‐US $75,000, or >US $75,000 per year), employment status, and smoking status were collected by self-report from the parent REGARDS study. We conceptualize race in this study as a social construct, reflective of the different social conditions in which people live in the United States [24-26].

### Data Collection Procedures

#### Cardinal LifeSpace App

The Cardinal LifeSpace data collection mobile app was developed in-house at Stanford University on the CardinalKit platform, an open-source, standards-based mobile development framework [27]. The platform included a Health Insurance Portability and Accountability Act (HIPAA)–ready cloud service with in-flight and at-rest encryption and fine-grained access control. Data were structured using HL7 FHIR and Open mHealth/IEEE 1752 standards, consistent with 21st Century Cures Act interoperability requirements. Account information was stored separately from study data, and all data were maintained in a deidentified format on a Research IT mobile health (mHealth) platform approved for protected health information. Participants were provided with a privacy policy during the consent process that could also later be accessed in the app during the study period.

Study participants were given a code and instructed to download the app. Participants engaged in the study for 2 weeks, after which they were instructed to delete the app from their phones. The app passively recorded latitude and longitude coordinates whenever the participant was in motion, captured at a spatial resolution of 500 m instead of at fixed temporal intervals. This sampling strategy prioritized the capture of significant spatial transitions over stationary or minor movements, in addition to preserving phone battery life. This tracking was active only while the device was powered on and remained subject to ongoing participant consent. If the app was opened, a map was displayed for the participant to see their location, and they had the option of turning off location tracking temporarily for privacy reasons. Each evening, participants were given a local notification and invited to participate in a survey asking 3 questions: (1) “How would you rate your day?” (sliding bar ranging from poor [1] to excellent [5]); (2) “How would you rate the environments in which you spent your time?” (sliding bar ranging from poor [1] to excellent [5]); and (3) “Is this map of your daily activity accurate?” (yes or no); if the participant answered no, a text box was displayed with the question, “Why?” Our app is currently only available for iPhone users, although we have plans to expand it to other platforms.

#### GPS Data Processing

To derive life-space metrics, mobile GPS data underwent several preprocessing steps. After importing GPS data, time stamps were converted from epoch time to the participants’ local time using the longitude of the GPS point. To capture the complete extent of the life space, spatial outliers were excluded. Data collected after 14 days were truncated. Participants’ home location was estimated by extracting the first GPS point recorded after 3 AM, rounding the latitude and longitude coordinates to the nearest 10th of a degree, and selecting the most frequently occurring rounded coordinate pair across the study period as the home location. Participants were included in the analysis if they had GPS data on at least 5 of the 14 observation days, with a valid day defined as having at least one GPS coordinate recorded.

#### Life-Space Metrics

**Figure 1.**
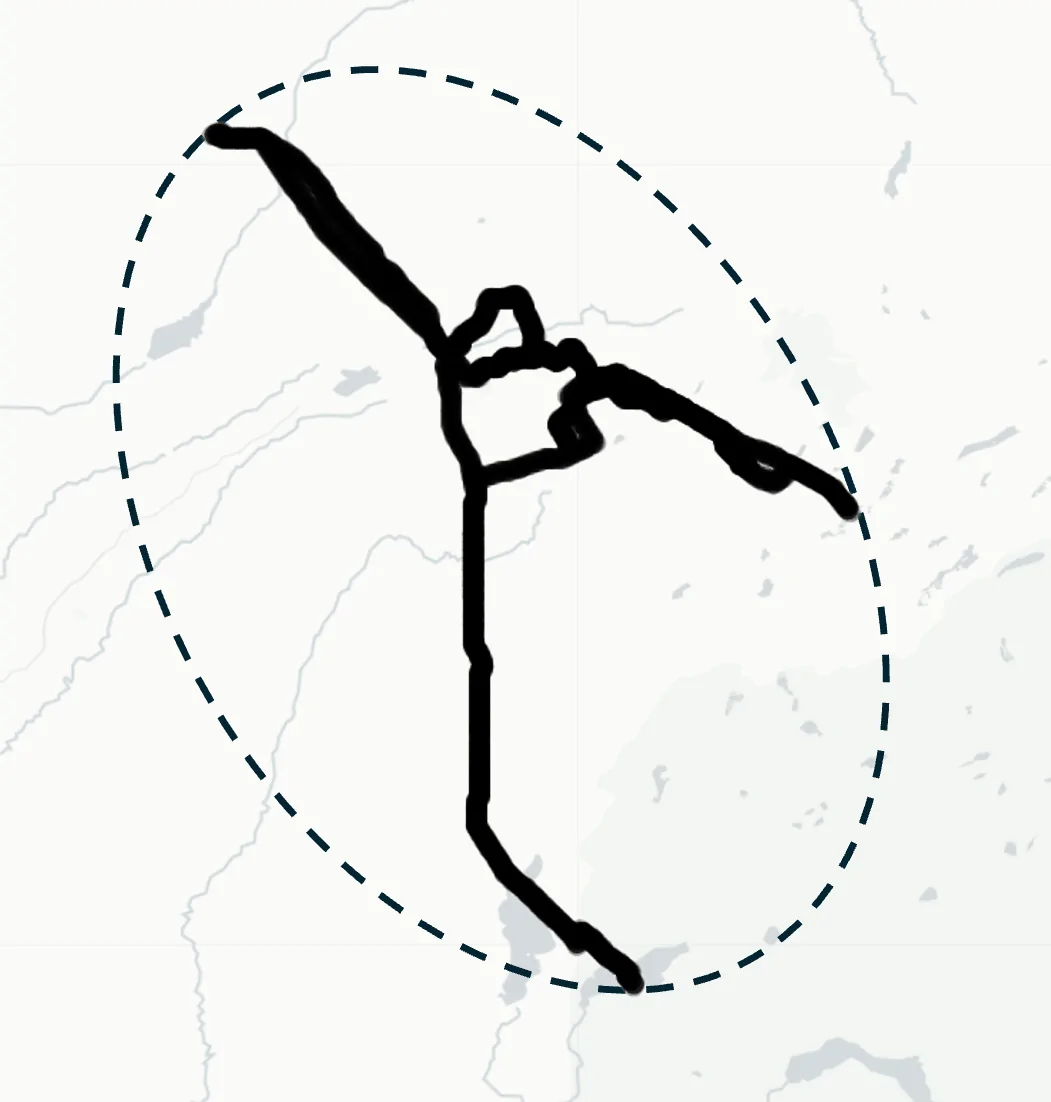
Representative deidentified life-space figure and overlaid metrics. GPS points from an example participant have been plotted and overlaid onto a random area of the United States, with scale maintained. The solid line represents the total distance traveled, and dashed line represents the ellipse area, defined as the area of the minimum ellipse encompassing all GPS points.

Participants’ life spaces were summarized from GPS coordinates as both daily average and total over the 2-week study period. We calculated life-space size using three GPS-derived metrics that were previously found to be associated with health outcomes: (1) ellipse area (km^2^) [4,28,29] (visualized in Figure 1), (2) total distance traveled across all GPS tracts (km) [28], and (3) maximum distance from home traveled over 2 weeks (km) [30,31]. Ellipse area was calculated as the area of the minimum ellipse that encompassed all points in 2 dimensions. Total distance provided an overall view of life-space size by summing the sequential distance between consecutive GPS points. Maximum distance from home was calculated as the greatest straight-line distance between each GPS observation and the estimated home location.

#### Street View Assessment

GSV images were retrieved from participants’ life spaces using GPS coordinates captured at a spatial resolution of 500 m. GSV images were downloaded according to the participant’s location using the Google Way package [32]. In order to create a panoramic image, images were downloaded and merged from 4 orientations (90**°** intervals). Sampling was contingent upon availability; if no imagery existed at the designated latitude and longitude, the point was bypassed. Images were run through the K-Net image segmentation algorithm, and features in the following categories were labeled: green space, sidewalks, benches, streetlights, and houses [33]. These 5 built environment features were selected because they support walkability and physical activity. The proportion of the image scored in each of these categories was summed and averaged, and each person was given a score for each feature.

### Statistical Analysis

We evaluated characteristics of participants who were screened versus enrolled in the pilot study and tested for differences using a *t* test for age and a chi-square test for all other variables. We additionally described the distribution of the life-space metrics and self-reported variables. The within- and between-person variances were calculated using an intercept-only linear random effects model of the self-reported variables; this model included a random intercept for each person and accounted for the fact that multiple ratings from the same individual may be correlated [34]. Differences in summary life-space metrics across participant characteristics were tested using the Wilcoxon rank sum test. In the supplementary analyses, we used a nonparametric repeated measures test, the aligned rank transform ANOVA, to test for differences in day and environmental ratings between groups [35].

The street view metrics were standardized by subtracting the mean and dividing by the SD. We tested for differences in the street view metrics by participant characteristics using the Wilcoxon rank sum test. We evaluated the correlation of the street view metrics using pairwise Pearson correlation coefficients.

All geospatial and statistical analyses were conducted using R (version 4.2.3; R Foundation for Statistical Computing) [36].

### Ethical Considerations

This study was approved by the Stanford University Institutional Review Board (IRB-65276) and the University of Alabama at Birmingham Institutional Review Board (IRB-020925004). After inviting people to participate in the study, participants were informed of the study goals, risks, and benefits through the mobile app, and they also provided informed consent per the approved protocol. Consent forms were stored separately from study data, and all data were maintained in a deidentified format on a Research IT mHealth platform approved for protected health information. Participants could receive up to US $100 for participation by completing all 14 nightly surveys and the close-out survey. Those who completed fewer surveys received less. No identification of individual participants in any images of the manuscript or supplementary material is possible.

### Reporting Framework

This paper was written following the STROBE (Strengthening the Reporting of Observational Studies in Epidemiology) guidelines (Checklist 1) for cross-sectional studies [37].

## Results

### Recruitment and Participant Flow

Recruitment and enrollment into the current study took place over 3 waves from June 2023 through January 2024. Among 1768 participants screened, 795 (45%) had an iPhone (Figure S1 in Multimedia Appendix 1). Among these participants, 538 (67.7%) said that they were possibly interested in a mobile phone study. There were 389 (72.3%) participants who met the eligibility criteria. Among the 389 eligible individuals, only 120 (30.8%) responded to our invitation letter, and 82 (68.3%) enrolled in the study.

### Participant Characteristics

The characteristics of those who did and did not participate are reported in Table 1. Overall, participants were younger, more likely to be female, and to identify as White. Participants had higher education, greater income, and were more likely to be employed compared with nonparticipants.

**Table 1.** Characteristics of participants enrolled in a study to assess life space versus those who were not enrolled from the REGARDS (Reasons for Geographic and Racial Differences in Stroke) study in the United States from 2023 to 2024.

	Nonparticipants (n=1686)	Participants (n=82)	*P* value^a^
Age (y), mean (SD)	78.83 (7.28)	74.54 (6.48)	<.001
White, n (%)	1099 (65.2)	68 (82.9)	.001
Men, n (%)	658 (39)	34 (41.5)	.75
Education level, n (%)			.002
College graduate and above	830 (49.2)	58 (70.7)	
High school graduate	339 (20.1)	9 (11)	
Less than high school	67 (4)	1 (1.2)	
Some college	450 (26.7)	14 (17.1)	
Urban group, n (%)			.56
Mixed (25%‐75% urban)	199 (11.9)	8 (10)	
Rural (≤25% urban)	222 (13.3)	8 (10)	
Urban (≥75% urban)	1248 (74.8)	64 (80)	
Income (US $), n (%)			<.001
<35,000	519 (32.2)	12 (15)	
35,000‐75,000	643 (39.9)	25 (31.2)	
>75,000	451 (28)	43 (53.8)	
Employment, n (%)			<.001
Employed	422 (25.4)	39 (48.1)	
Retired	1101 (66.2)	36 (44.4)	
Other	141 (8.5)	6 (7.4)	

a Differences between groups were tested using a *t* test for age and chi-squared test for all other variables.

### Statistics and Data Analysis

Among 82 enrolled participants, 76 (92.7%) had 14 days of GPS data collected. Since all participants had at least 5 days with GPS data recorded, we did not exclude any participants from the analysis, and there were no missing data on covariates. The median number of days of response to the nightly questionnaires was 14 (IQR 11-14; range 2-14). There was a high degree of variability across participants in the life-space metrics (Table 2). For example, the total distance traveled over 2 weeks ranged from 39 km to 31,743 km. Participants rated the overall quality of their day as “very good” (median 4) and their environment as “excellent” (median 5), and the within-person variability over 14 days approximated the between-person variability of these measures.

**Table 2.** Distribution of life-space metrics and self-reported ratings of day and environment from poor (rating: 1) to excellent (rating: 5) in participants recruited from the REGARDS (Reasons for Geographic and Racial Differences in Stroke) study in the United States from 2023 to 2024.

	Ratings, median (IQR; range)	Within-person SD	Between-person SD
Ellipse area (km^2^)	77.4 (17.6-308.8; 0.189-3,100,917)	—^a^	—
Maximum distance from home (km)	29.5 (16.8-78.6; 2.54-5074.9)	—	—
Total distance traveled (km)	477.1 (234.4-817.1; 39.05-31743.11)	—	—
Day rating	4 (4-5; 3-5)	0.65	0.55
Environmental rating	5 (3-5; 2-5)	0.65	0.59

a Not applicable.

On average, participants younger than 70 years, female participants, and Black participants had larger life space measured by the life-space ellipse than those aged 70 years and older, male participants, and White participants (154.5 vs 58.1 km^2^, *P*=.04; 102.9 vs 46.0 km^2^, *P*=.07; 179.3 vs 62.8 km^2^, *P*=.03; and 145.5 vs 25.5 km^2^, *P*=.001), although not all of these comparisons met the α=.05 level of statistical significance based on the Wilcoxon rank sum test (Table 3). Female participants were likely to travel a farther maximum distance from home than male participants (31.3 km vs 23.6 km, *P*=.02), and younger participants had a greater total distance traveled compared with older participants (561.7 vs 410.5 km, *P*=.08), although this difference did not meet the α=.05 level of statistical significance based on the Wilcoxon rank sum test. Of the socioeconomic variables, higher income (>US $75,000 per year) was associated with higher life space, maximum distance from home, and a greater total distance traveled compared with <US $75,000 per year (145.5 vs 25.5 km^2^, *P*=.001; 40.3 vs 18.0 km, *P*=.008; and 576.5 vs 320.0 km, *P*=.004, respectively). The daily ratings and environmental ratings did not differ by age, gender, or race (*P*>.05 for all comparisons; Table S1 in Multimedia Appendix 1).

**Table 3.** Median life-space measures by participant characteristics. Participants were recruited from the REGARDS (Reasons for Geographic and Racial Differences in Stroke) study in the United States from 2023 to 2024.

	Ellipse (km^2^), median (IQR)	*P* value	Max distance from home (km), median (IQR)	*P* value	Total distance traveled (km), median (IQR)	*P* value
Age (y)		.04		.17		.08
<70	154.5		35.2		561.7	.08
>70	58.1		28.9		410.5	
Gender		.07		.02		.12
Women	102.9		31.3		499.4	
Men	46.0		23.6		343.4	
Race		.03		.11		.08
Black	179.3		40.6		577.9	
White	62.8		27.3		391.9	
Education		.87		.73		.56
College	78.9		29.7		482.4	
Less than college	58.3		23.6		409.1	
Income (US $)		.001		.008		.004
>75,000	145.5		40.3		576.5	
≤75,000	25.5		18.0		320.0	
Employment		.26		.77		.33
Currently employed	125.6		30.1		493.9	
Not currently employed	62.9		28.9		446.3	

The street view metrics were strongly intercorrelated. Greenness was associated with lower levels of sidewalk (ρ=−0.32; *P*=.003) and higher levels of streetlight (ρ=0.50; *P*<.001) and house (ρ=0.48; *P*<.001). The sidewalk variable was associated with greater levels of streetlights (ρ=0.62; *P*<.001) and benches (ρ=0.89; *P*<.001) but lower measured occurrences of houses (ρ=−0.49; *P*<.001). The bench and streetlight variables were positively correlated (ρ=0.70; *P*<.001), while houses and benches were negatively correlated (ρ=−0.60; *P*<.001; Table S2 in Multimedia Appendix 1). Street view metrics were characterized by demographic groups (Table S3 in Multimedia Appendix 1). Overall, metrics were similar across age, gender, race, education, income, and employment categories, although women had slightly higher exposure to green space than men, those with a college education had slightly lower bench and greater house values than those without a college education, and those currently employed had slightly lower streetlight and bench values than those not currently employed.

## Discussion

### Principal Findings

In this feasibility study, we demonstrated that participants’ personal mobile phones can be used to passively track their life space. Among 82 older adult participants, we found that participants younger than 70 years, female participants, Black participants, and participants with higher income had a larger life space compared with those 70 years and older, male participants, White participants, and those with lower income. We additionally were able to capture features of the physical environment from GSV imagery. The participants in our study were a selected population of REGARDS participants, and future studies should aim to streamline enrollment to lower the bar to participation.

Our study built upon other recent work using GPS technologies to capture life-space and mobility patterns among older adults [12]. In a 2021 systematic review of 29 studies using GPS tracking technology to measure mobility, only one of the studies used the participants’ smartphone devices [12]. Although location tracking is common among commercial smartphone apps, the uptake in research settings appears to lag behind, with only a small number of investigations using GPS tracking in commercial devices in older adults [10,11]. This may be due to time and technology barriers to app development. We used an open-source framework for digital health apps and research that was developed to accelerate the prototyping of digital health apps [38]. This set of tools enabled us to develop a relevant app from the ground up in a reasonably short time while ensuring that the app met our standards for security and accessibility, as opposed to starting from scratch, which would have slowed development.

Consistent with the literature summarized in the 2021 systematic review, we found that life-space mobility was smaller among older adults [12]. In contrast to prior research, we found that women traveled a greater distance from home relative to men [12,39]. The reason for this difference is unclear and could be due to chance. In our population, Black participants had a greater life space compared with White participants. This may be due to the higher prevalence of employment among Black participants in our study or other factors including social connections and leisure activities. We found that participants with higher income had higher life space, greater maximum distance traveled, and greater total distance traveled, and this was consistent with prior literature demonstrating that low-income adults spend a greater proportion of time at home and have smaller life-space measures [12,29]. This may be due to the greater available resources that higher income affords, including access to transportation and money to pay for gas, food, and activities outside the home.

Our findings demonstrate that passive smartphone-based life-space monitoring is feasible and well-accepted in research studies. Additionally, this technology could support the care of older adults by enabling early detection of restricted life space without patient burden, potentially triggering follow-up to investigate and address the root causes [6]. The observed differences by income and race highlight how this technology could identify populations who may benefit from targeted interventions such as improved transportation access or community resources [6]. Furthermore, linking mobility patterns with environmental data could inform age-friendly urban planning and identify modifiable built environment features that promote or restrict older adults’ real-world movement and independence [9].

As a feasibility study, our study was necessarily limited in scope due to time and resource constraints, and several limitations should be considered when interpreting the findings. Our study population represented a selected sample of REGARDS participants who responded to a mailed recruitment letter, limiting generalizability to the broader REGARDS cohort and other community-dwelling adults. Specifically, our participants were younger and had greater education and income, which may correlate with greater technological literacy, health engagement, and economic resources. Following REGARDS ancillary study protocols, eligible participants were invited to participate by a letter. Email-based recruitment could enable faster response times and communication. Moreover, in-person enrollment during existing study visits would substantially lower barriers to participation, particularly for individuals with limited technological literacy or confidence. We recommend that future studies use this approach, when feasible, to improve generalizability. Additionally, we recommend having dedicated study personnel, ideally at an in-person visit, to enroll study participants and facilitate downloading and installing the mobile app. When in-person support is not feasible, participants should have access to study staff via telephone or email during extended business hours. Due to funding constraints, we were unable to staff our helpline continuously and thus needed to return phone calls from participants asynchronously, which slowed down recruitment and disadvantaged those with limited flexibility. We anticipate that technological support needs will attenuate over time as digital literacy increases among older adults. Nonetheless, ensuring accessible technical assistance remains critical for equitable participation across diverse populations, particularly among those with limited prior smartphone experience, lower educational attainment, or cognitive impairment. Finally, our app was designed to maximize battery efficiency by sampling GPS coordinates only every 500 m, but this constraint limited our ability to capture microscale environmental variations, particularly in dense urban environments.

### Conclusions

This first-generation study is an important demonstration of the feasibility of integrating life-space assessment and mobility measures into older adults’ daily routine through the use of a smartphone app. Unlike traditional questionnaire-based methods to assess life space, this approach can offer real-world continuous assessment and is not susceptible to recall bias. An additional strength of this approach is that it adds virtually no additional measurement burden to the participant, and thus could be used to capture life space over an extended period of weeks, months, or years. Integration of a life-space assessment into older adults’ daily lives may allow for the use of life space as an integrative health metric, and changes in life space could be studied for their prognostic value. New open-source platforms can accelerate time to development for digital health apps, thus supporting the feasibility of smartphone apps in population health research.

## Supplementary material

10.2196/73128 Multimedia Appendix 1 REGARDS (Reasons for Geographic and Racial Differences in Stroke) participant recruitment (2023-2024, N=82), environmental ratings, and Google Street View life-space metrics.

10.2196/73128 Checklist 1 STROBE checklist.

## Abbreviations

GSV: Google Street View
mHealth: mobile health
REGARDS: Reasons for Geographic and Racial Differences in Stroke
STROBE: Strengthening the Reporting of Observational Studies in Epidemiology
