# Supplementary material for: Objectively Measured Life Space Using Participants’ Mobile Phones: Cross-Sectional Study
Source: JMIR Form Res. 2026 Sep 21;10:e73128. doi: 10.2196/73128 (PMC13592664; doi:10.2196/73128)
Supplement: Supplementary file 2 [file formative-v10-e73128-s001.docx]

Supplemental Figure 1: Consort figure depicting the recruitment and enrollment of participants from the REGARDS study in the United States. Participants were recruited between June 2023 and January 2024. A total of 82 participants were eligible, responded to the mailed study invitation, and chose to enroll in the study.

Supplemental Table 1: Day and environmental ratings, from poor (1) to excellent (5), by Participant Characteristics. Participants were recruited from the REGARDS study in the United States from 2023-2024.

|  |  | Median Day Rating | *P*-value | Median Environmental Rating | *P*-value |
| --- | --- | --- | --- | --- | --- |
| Age | Age <70 years | 4.0 | .27 | 4.0 | .33 |
|  | Age 70+ years | 5.0 |  | 5.0 |  |
| Sex | Female | 4.0 | .88 | 5.0 | .81 |
|  | Male | 4.0 |  | 5.0 |  |
| Race | Black Race | 4.0 | .48 | 4.0 | .93 |
|  | White Race | 4.0 |  | 5.0 |  |

* An aligned rank transform ANOVA revealed no difference in ratings by age, sex, or race.

Supplemental Table 2: Correlation of street view metrics as assessed in participants’ life-space. Participants were recruited from the REGARDS study in the United States from 2023-2024.

|  | Greenness | Sidewalk | Streetlight | Bench | House |
| --- | --- | --- | --- | --- | --- |
|  | **Rho**  *P*-value | | | | |
| Greenness | **1.00** | * | * | * | * |
| Sidewalk | **-0.32**  .003 | **1.00** | * | * | * |
| Streetlight | **0.50**  <.001 | **0.62**  <.001 | **1.00** | * | * |
| Bench | **-0.10**  .40 | **0.89**  <.001 | **0.70**  <.001 | **1.00** | * |
| House | **0.48**  <.001 | **-0.49**  <.001 | **-0.03**  .81 | **-0.60**  <.001 | **1.00** |

|  | Green Space* | *P*-value | Sidewalk* | *P*-value | Streelight* | *P*-value | Bench* | *P*-value | House* | *P*-value |
| --- | --- | --- | --- | --- | --- | --- | --- | --- | --- | --- |
| **Age** |  |  |  |  |  |  |  |  |  |  |
| Age <70 years | 4.27 | .32 | 2.40 | .93 | 2.89 | .65 | 1.24 | .97 | 1.33 | .59 |
| Age 70+ years | 4.07 |  | 2.56 |  | 2.79 |  | 1.18 |  | 1.48 |  |
| **Sex** |  |  |  |  |  |  |  |  |  |  |
| Female | 4.31 | .05 | 2.50 | .82 | 2.96 | .26 | 1.18 | .68 | 1.58 | .21 |
| Male | 3.87 |  | 2.53 |  | 2.63 |  | 1.23 |  | 1.23 |  |
| **Race** |  |  |  |  |  |  |  |  |  |  |
| Black Race | 4.20 | .42 | 2.89 | .07 | 3.25 | .16 | 1.54 | .13 | 1.39 | .84 |
| White Race | 4.11 |  | 2.44 |  | 2.73 |  | 1.13 |  | 1.44 |  |
| **Education** |  |  |  |  |  |  |  |  |  |  |
| College | 4.19 | .74 | 2.84 | .10 | 2.78 | .78 | 1.03 | .005 | 1.62 | .007 |
| Less than college | 3.97 |  | 2.38 |  | 2.92 |  | 1.60 |  | 0.97 |  |
| **Income** |  |  |  |  |  |  |  |  |  |  |
| Income>$75K | 4.16 | .63 | 2.41 | .30 | 2.75 | .59 | 1.10 | .30 | 1.52 | .38 |
| Income<=$75K | 4.09 |  | 2.63 |  | 2.90 |  | 1.30 |  | 1.34 |  |
| **Employment** |  |  |  |  |  |  |  |  |  |  |
| Currently employed | 4.09 | .78 | 2.29 | .07 | 2.55 | .04 | 0.96 | .008 | 1.41 | .88 |
| Not currently employed | 4.17 |  | 2.72 |  | 3.07 |  | 1.42 |  | 1.45 |  |

Supplemental Table 3: Median value of Google Street View metrics by Study Characteristics participant characteristics. Participants were recruited from the REGARDS study in the United States from 2023-2024.

*Street View metrics were standardized and differences in street view metrics between groups was assessed using the Wilcoxon rank sum test.
